# A Cell Culture System to Investigate Marek’s Disease Virus Integration into Host Chromosomes

**DOI:** 10.3390/microorganisms9122489

**Published:** 2021-12-01

**Authors:** Yu You, Tereza Vychodil, Giulia Aimola, Renato L. Previdelli, Thomas W. Göbel, Luca D. Bertzbach, Benedikt B. Kaufer

**Affiliations:** 1 Institute of Virology, Freie Universität Berlin, 14163 Berlin, Germany; yuyou@zedat.fu-berlin.de (Y.Y.); tereza.vychodil@fu-berlin.de (T.V.); giulia.aimola@fu-berlin.de (G.A.); rprevidelli@rvc.ac.uk (R.L.P.); 2 Department of Comparative Biomedical Sciences, Royal Veterinary College, London NW1 0TU, UK; 3 Institute for Animal Physiology, Department of Veterinary Sciences, Ludwig Maximilian University Munich, 80539 Munich, Germany; goebel@lmu.de; 4 Department of Viral Transformation, Leibniz Institute for Experimental Virology (HPI), 20251 Hamburg, Germany

**Keywords:** MDV, herpesvirus, telomere integration, in vitro assay, telomeric repeats, fluorescence in situ hybridization, genome maintenance, latency, viral transformation

## Abstract

Marek’s disease virus (MDV) is a highly oncogenic alphaherpesvirus that causes a devastating neoplastic disease in chickens. MDV has been shown to integrate its genome into the telomeres of latently infected and tumor cells, which is crucial for efficient tumor formation. Telomeric repeat arrays present at the ends of the MDV genome facilitate this integration into host telomeres; however, the integration mechanism remains poorly understood. Until now, MDV integration could only be investigated qualitatively upon infection of chickens. To shed further light on the integration mechanism, we established a quantitative integration assay using chicken T cell lines, the target cells for MDV latency and transformation. We optimized the infection conditions and assessed the establishment of latency in these T cells. The MDV genome was efficiently maintained over time, and integration was confirmed in these cells by fluorescence in situ hybridization (FISH). To assess the role of the two distinct viral telomeric repeat arrays in the integration process, we tested various knockout mutants in our in vitro integration assay. Efficient genome maintenance and integration was thereby dependent on the presence of the telomeric repeat arrays in the virus genome. Taken together, we developed and validated a novel in vitro integration assay that will shed light on the integration mechanism of this highly oncogenic virus into host telomeres.

## 1. Introduction

Marek’s disease is one of the most important and widespread infectious diseases in chickens, causing great economic losses in the poultry industry worldwide [1,2]. The causative agent of this lymphoproliferative disease is Marek’s disease virus (MDV). This virus causes major losses in the poultry industry, despite widespread vaccination for decades and extensive research on MDV virulence factors, host resistance and more efficient vaccines. This is mainly due to the increase in MDV virulence that has occurred over recent decades [3,4,5]. During primary infection, MDV productively replicates in antigen-presenting cells and lymphocytes [6,7,8]. Upon infection of the host, MDV establishes latency primarily in CD4+ T cells, allowing the virus to persist in the host for life [9]. These infected CD4+ T cells can also become transformed, resulting in deadly lymphomas [10]. Both latently infected and MDV-induced tumor cells harbor the integrated virus genome in the telomeres of one or multiple host chromosomes [11,12,13]. Intriguingly, several other herpesviruses are also able to integrate their genome into host telomeres [14,15]. We previously demonstrated that the integration of MDV and human herpesvirus 6 (HHV-6) is facilitated by telomeric repeat arrays (TMRs) present at both ends of their linear genomes [12,16]. These TMRs consist of hexanucleotide (TTAGGG)_n_ repeats that are identical to the telomere sequences in all vertebrates [14,17]. The MDV genome contains two distinct TMR arrays, a stretch of multiple telomeric repeats (mTMR) with a variable number of repeats, and short telomeric repeats (sTMR) with a fixed number of six repeats. Until now, MDV integration could only be investigated in vivo in a qualitative manner due to the lack of an in vitro integration assay.

Therefore, we set out to establish a cell culture-based system to quantitatively evaluate MDV integration without the need for laboratory animals. First, we tested several chicken T cell lines for their ability to facilitate MDV latency, genome maintenance and integration. Based on this information, we developed a quantitative integration assay that was used to investigate the integration efficiencies of wild type and mutant viruses lacking either mTMR, sTMR or both. This quantitative integration system provides an optimal basis for investigating the role of viral and cellular factors in the integration of MDV into the host telomeres.

## 2. Materials and Methods

### 2.1. Cells

The reticuloendotheliosis virus (REV)-transformed chicken T cell lines CU91, IV A5, 855-19 and 855-23 [18,19], were cultured in RPMI 1640 (PAN Biotech; Aidenbach, Germany) supplemented with 1% sodium pyruvate (PAN Biotech), 1% non-essential amino acids (Biochrom; Berlin, Germany), 10% fetal bovine serum (PAN Biotech) and 1% penicillin (100 U/mL)/streptomycin (100 µg/mL) (AppliChem; Darmstadt, Germany) and maintained at 41 °C in a 5% CO_2_ atmosphere. Chicken embryo cells (CEC) were generated from Valo specific-pathogen-free (SPF) embryos (VALO BioMedia GmbH, Osterholz-Scharmbeck, Germany) and maintained as described previously [20].

### 2.2. Generation of Mutant Viruses

**Figure 1:**
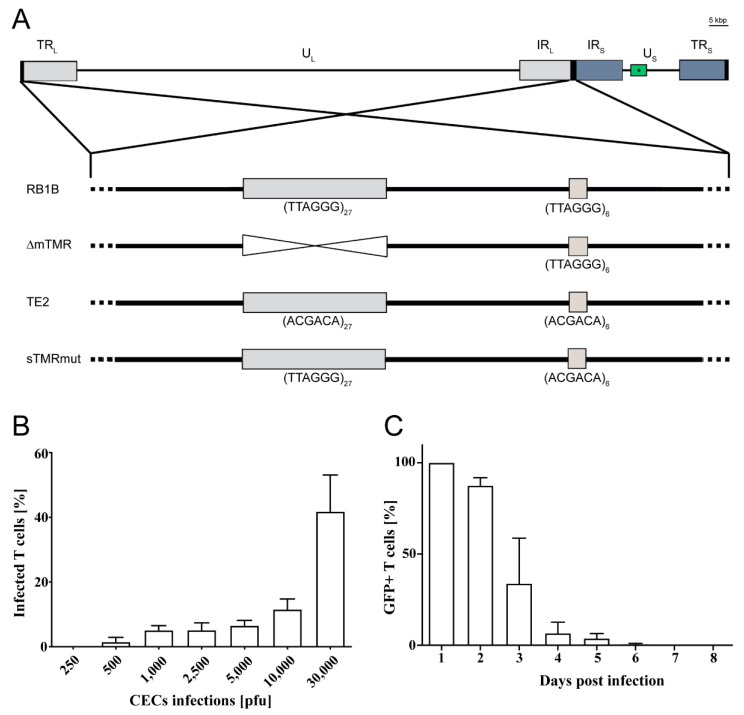
Recombinant viruses and infection of T cells. (**A**) Schematic overview of the MDV genome containing its two unique regions, unique long (UL) and short (US) regions, that are flanked by terminal (TRL and TRS) and internal (IRL and IRS) inverted repeat regions. Modifications made to the mTMR and sTMR within the a-like sequences located in the terminal repeat (TR) and internal repeat (IR) region of the MDV genome are shown for the indicated mutant viruses. (**B**) Infection of one million CECs with indicated virus doses. CU91 T cells were seeded on the infected monolayer for 16 h, and the number of infected GFP+ T cells was subsequently quantified by FACS. (**C**) Assessment of GFP expression in infected FACS-sorted T cells at indicated time points after infection. Shown are the mean values of 3 independent experiments. The error bars indicate the standard deviation.

Recombinant viruses were generated using pRB1B, an infectious bacterial artificial chromosome (BAC) clone of the highly oncogenic RB1B MDV strain (GenBank accession no. MT797629) [21], using two-step Red-mediated mutagenesis as described previously [12,22,23]. To visualize infected cells, an enhanced green fluorescent protein (eGFP) expression cassette driven by the HSV-1 thymidine kinase (TK) promoter was inserted into the minimal fertility factor (mini-F) of the wild type and previously generated viral telomere mutants: ΔmTMR containing a complete deletion of the mTMRs (TTAGGG)27; sTMRmut in which the sTMR repeats (TTAGGG)_6_ were replaced by scrambled repeats (ACGACA)_6_; and TE2 in which both the sTMR and mTMR were replaced by scrambled repeats (ACGACA)_n_ (Figure 1) [12,24]. Briefly, a universal transfer construct harboring TK-GFP and a positive selection marker (I-SceI-aphAI) was generated. The TK-GFP-I-SceI-aphAI cassette was amplified using primers containing homologous sequences for recombination (Table 1) [25]. The purified PCR product was introduced into GS1783 *E. coli* harboring pRB1B or the respective telomere mutants. Positive clones were selected and screened by restriction fragment length polymorphism (RFLP) analysis. Upon removal of the positive selection, all clones were confirmed by RFLP, PCR and Sanger sequencing of the targeted region. Recombinant viruses were reconstituted by transfection of CEC with purified BAC DNA using calcium phosphate transfection as described previously [26]. All viruses were propagated in CEC. Virus stocks were frozen in liquid nitrogen and titrated on fresh CEC.

### 2.3. T Cell Infection

Chicken T cell lines were infected by seeding them onto an infected CEC monolayer [27]. For that, one million CECs were infected with 250–30,000 plaque-forming units (pfu) of cell-associated GFP-reporter viruses (RB1B wild type and telomere mutant viruses) as indicated in 6-well plates for 4 days. Subsequently, one million T cells per well were added to the highly infected CEC monolayer for 16 h at 41 °C. T cells were then carefully removed by pipetting and either analyzed or sorted by FACS.

### 2.4. Quantification of MDV Genome Copy Numbers by qPCR

DNA was isolated from cells using the RTP DNA/RNA Virus Mini Kit (Stratec; Berlin, Germany) according to the manufacturer’s instructions. MDV genome copies were determined by quantitative PCR (qPCR) using specific primers and a probe for the ICP4 gene (MDV084). ICP4 copy numbers were normalized against the genome copies of cellular inducible nitric oxide synthase (iNOS), as described previously [28] (Table 1).

### 2.5. RT-qPCR

RNA was isolated from MDV-infected 855-19 T cells at different time points post-infection using the RNeasy Plus Mini Kit (Qiagen, Hilden, Germany). The isolated RNA was treated with DNase I (Promega, Fitchburg, WI, USA), and cDNA was synthesized using a High-Capacity cDNA reverse transcription kit (Thermo Fisher, Waltham, MA, USA). Expression levels of UL36, pp38 and vTR were measured by RT-qPCR and normalized against cellular glyceraldehyde-3-phosphate dehydrogenase (GAPDH), as previously described (Table 1) [29,30].

### 2.6. Flow Cytometry

A fraction of infected T cells was stained with propidium iodide for viability assessment and GFP expression kinetics. Data were analyzed with the CytoFlex S FACS analyzer (Beckman Coulter, Brea, CA, USA) and evaluated using CytExpert Software.

### 2.7. Fluorescence In Situ Hybridization

The metaphase chromosomes were prepared from infected T cells on day 14 post-infection (dpi) and analyzed for the presence of the MDV genome by FISH [24,31]. Briefly, MDV genomes were detected using a set of PCR-based MDV probes that were generated using the Biotin PCR Labeling Kit (PromoCell, Heidelberg, Germany) (for primers, see Table 1). Virus genomes were visualized using Cy3 Streptavidin (1:1000; GE Healthcare, PA43001; Munich, Germany), metaphase FISH images were taken using an Axio Imager M1 system and the AxioVision software (Carl Zeiss, Inc.; Oberkochen, Germany) and analyzed with ImageJ (https://imagej.nih.gov/ij/, accessed on 28 November 2021). Appropriate positive and negative controls were included (Figure S2).

### 2.8. Reactivation

MDV reactivation from latently infected 855-19 cells was induced 14 dpi by incubation at room temperature for 30 min and serum starvation throughout the co-cultivation period, as described previously [32]. Briefly, for each virus mutant, 10,000 treated T cells were seeded onto a confluent CEC monolayer and carefully washed off 24 h post-seeding. The reactivation efficiency for each mutant virus was measured by counting plaque on the CEC monolayer at 6 dpi.

### 2.9. Statistical Analyses

Statistical analyses were performed using GraphPad Prism version 8 (San Diego, CA, USA). qPCR results of MDV genome copies, as well as integration efficiencies, were analyzed using the Kruskal–Wallis and Mann–Whitney U tests, respectively. Results were considered significantly different when *p* < 0.05.

## 3. Results and Discussion

### 3.1. MDV Efficiently Infects CU91 T Cells

The REV-transformed CU91 chicken T cell line was previously established at Cornell University [18] and has been shown to be infectable with MDV [33,34]. To establish our in vitro integration assay, we first generated reporter viruses based on the very virulent RB1B strain (wild type and TMR mutant viruses) by inserting a GFP cassette driven by the TK promoter (Figure 1A). We then optimized the infection of CU91 T cells by seeding them on CEC monolayers infected with 250 to 30,000 pfu, since MDV is a highly cell-associated virus. Using 30,000 pfu, we consistently observed T cell infection rates of 40–50% with high viability (Figure 1B). Next, we assessed whether the virus genome is silenced over time in T cells by monitoring the loss of GFP expression in FACS-sorted infected T cells (Figure 1C). The GFP in the MDV genome was rapidly silenced in the infected cells, and no lytic replication was detected after 7 dpi. Moreover, viral lytic but not latent gene expression levels progressively decreased in infected T cells (Figure S1). This silencing of the MDV genome is consistent with previous studies by Parcells and colleagues [33,34].

### 3.2. MDV Genome Maintenance in Infected CU91 T Cells Is Dependent on Viral TMR

**Figure 2:**
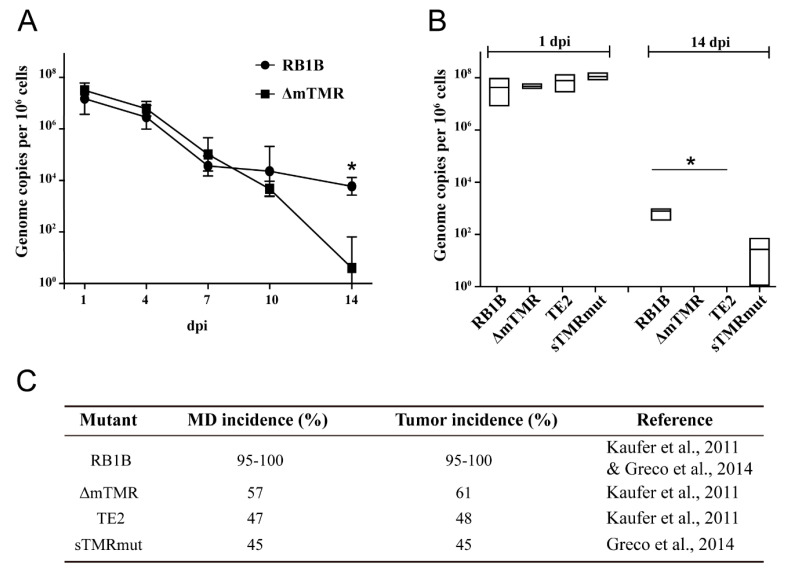
Genome maintenance of wt and mutant viruses in CU91 cells. (**A**) Monitoring viral genome copies in infected CU91 T cells over time with indicated viruses. Mean viral genome copies per million cells of 3 independent experiments are shown (* *p* < 0.05, Mann–Whitney U test). (**B**) Viral genome maintenance after CU91 T cell infections with indicated viruses were compared. Mean viral genome copies per million cells are shown as a box with the minimum and maximum. The asterisk indicates significant differences compared to RB1B (* *p* < 0.05, Kruskal–Wallis test, n = 3). (**C**) In vivo data previously published for the wt and TMR mutant viruses with respective references [12,24]. MD = Marek’s disease.

It has been previously shown that MDV establishes latency in CU91 T cells [33,34], suggesting that the virus likely integrates and is maintained in these cells. Therefore, we infected CU91 with the RB-1B wild-type (wt) virus and monitored virus genome levels over time. After initial virus replication in some of the cells resulted in high genome levels, lytic replication ceased, as observed in Figure 1C and Figure 2A. MDV genome copies remained relatively constant after 7 dpi, indicating that the virus is stably maintained in some of the cells (Figure 2A).

To determine if the viral telomeres contribute to MDV genome maintenance, we tested TMR deficient viruses that we previously evaluated in vivo [12,24]. While the wt virus was consistently maintained in the culture, a virus that lacks the mTMR repeat arrays (ΔmTMR), could not be detected at 14 dpi (Figure 2B). Similarly, maintenance of a virus in which both the mTMR and sTMR were replaced by a scrambled repeat sequence (TE2) was below the detection limit in the culture. Less efficient maintenance compared to the wt virus was also observed for a virus in which the sTMRs were replaced by scrambled repeats (sTMRmut; Figure 2B). These results are consistent with our previous in vivo studies, which revealed that integration efficiency and the ability to cause tumors of all three TMR mutant viruses was severely impaired in experimentally infected chickens (Figure 2C) [12,24]. Our data demonstrated that both mTMR and sTMR play a crucial role in the maintenance of MDV in CU91 T cells over time.

### 3.3. MDV Genome Maintenance Differs between Chicken T Cell Lines

**Figure 3:**
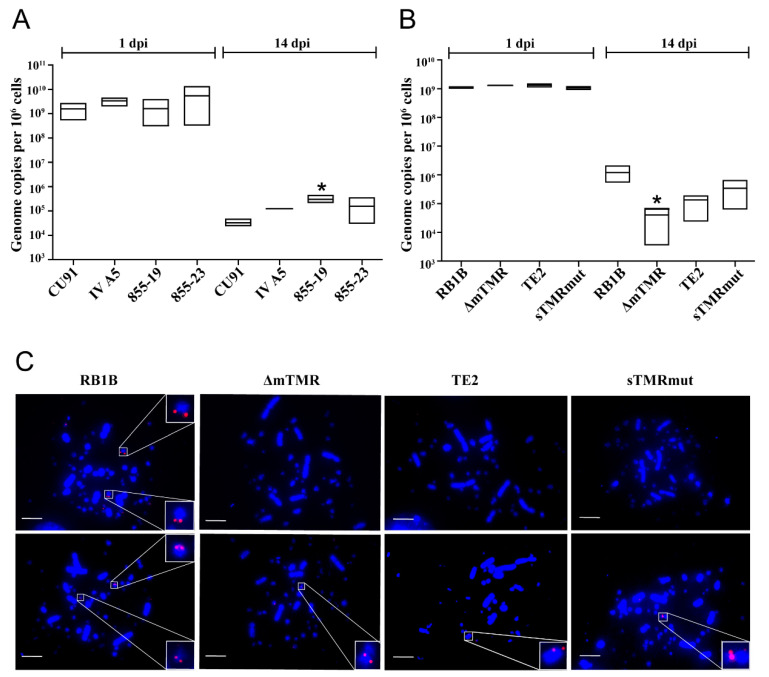
Genome maintenance and integration of the wt and mutant virus in T cells. (**A**) Evaluation of RB1B genome maintenance in different T cell lines. Mean viral genome copies per million cells are shown as box plots with the minimum and maximum. An asterisk indicates significant differences compared to CU91 T cell infections (* *p* < 0.05, Kruskal–Wallis test, n = 3). (**B**) Comparison of virus maintenance after 855-19 T cell infection with the indicated viruses. Significant differences are in comparison to RB1B (* *p* < 0.05, Kruskal–Wallis test, n = 3). (**C**) Representative metaphase chromosomes (DAPI stain, blue) are shown along with the presence and location of integrated MDV (Cy3 streptavidin, red) in 855-19 infections with RB1B wild-type and different TMR mutants as indicated. Two representative images per infection are shown. Scale bars correspond to 10 μm.

Even though the CU91 system provided exciting data on the integration efficiency of wt and mutant viruses, only a small proportion of cells maintained the wt virus. To assess virus integration by FISH and reactivation of the virus, a higher percentage of potential latently infected cells were needed. Therefore, we tested the efficiency of MDV genome maintenance in three additional REV-transformed T cell lines, IV A5, 855-19 and 855-23, following the CU91 T cell infection protocol. qPCR revealed that all three T cell lines are capable of maintaining the viral genome even at higher levels compared to CU91 (Figure 3A). The 855-19 T cell line provided the best genome maintenance and was therefore further used to assess MDV maintenance and integration of latent genomes.

### 3.4. Impaired Genome Maintenance and Integration of TMR Mutants in 855-19 T Cells

To assess the maintenance and integration of MDV in 855-19 T cells, we infected these cells with wt RB1B, ΔmTMR, TE2 and sTMRmut viruses. 855-19 T cells were able to support MDV genome maintenance at a higher level compared to CU91 T cells. In RB1B-infected 855-19 T cells at 14 dpi, we detected roughly one virus genome copy per cell by qPCR. As observed in CU91 cells, integration was severely impaired in the case of the mutant viruses ΔmTMR, TE2 and sTMRmut (Figure 3B). Genome maintenance was reduced by about 30-fold for ΔmTMR, 10-fold for TE2 and 4-fold for sTMRmut compared to the wt virus. These data are comparable to a previous study investigating the integration of human herpesvirus 6A (HHV-6A) in vitro, which revealed that two TMR arrays in the HHV-6A genome are required for efficient integration in human cells [16].

Since RB1B maintains its genome in latently infected and tumor cells by integrating its genome into host telomeres [11,12], we investigated 855-19 T cells infected with wt and mutant viruses by FISH after 14 dpi. FISH analysis revealed that RB1B integrated in one or multiple chromosomes of about 10% of the infected 855-19 T cells (Figure 3C). This integration was consistently observed at the end of the chromosomes, as previously shown for latently infected and tumor cells ex vivo. In contrast, we only detected very few integration events in cells infected with the TMR mutant viruses (Figure 3C). The chicken lymphoblastoid MDV-transformed cell line MSB-1 [35] and primary MDV-induced tumor cells were used as positive controls (Figure S2). These integrations were also not at the ends of the host chromosomes in many cases, a phenomenon consistent with previous findings that these viruses do not integrate into telomeres of tumor cells obtained from infected animals [12,14].

### 3.5. The MDV Genome Can Reactivate from Latency

**Figure 4:**
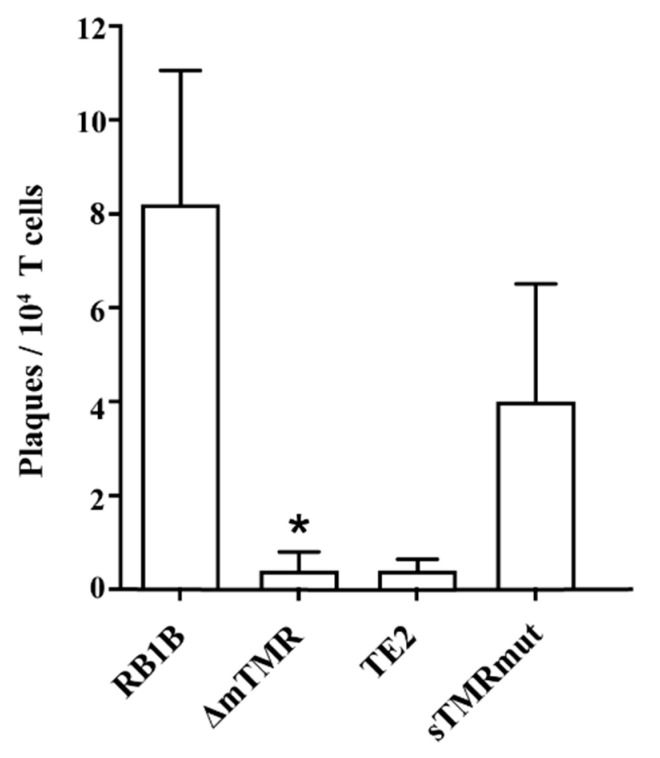
Reactivation efficiency in latently infected 855-19 T cells (14 dpi) infected with the indicated viruses. The data are shown as the mean number of plaques per 10,000 treated T cells on the CEC monolayer (* *p* < 0.05, Kruskal–Wallis test, n = 3). The error bars indicate the SEM.

Since MDV can reactivate from its integrated state [12,36], we set out to examine the reactivation properties of the integrated viruses in infected 855-19 T cells 14 dpi. Reactivation was induced by incubation of the cells at room temperature for 30 min, serum starvation, and seeding them onto a CEC monolayer for 24 h. After 6 days, plaques on CEC were counted. Reactivation of RB1B was readily observed by the formation of many plaques. The plaque number was decreased by approximately 10-fold more for ΔmTMR and TE2 and 2-fold for sTMRmut (Figure 4).

## 4. Conclusions

Until now, investigation of the MDV integration mechanism and viral factors involved in this process required animal experiments due to the lack of feasible cell culture-based assays. Previous studies showed that viral telomeric repeats (TMRs) facilitate MDV integration into chicken telomeres in vivo. Upon deletion of the viral TMRs, disease and tumor formation were severely impaired in infected chickens. In addition, the number of genomes per tumor cell was reduced to a single concatemeric genome that was not located in host telomeres [12].

In this study, we established a quantitative in vitro integration assay using immortalized chicken T cell lines, the target cells for MDV latency and transformation. This assay provides a crucial platform for the analysis of the integration mechanisms. It could be used to study the role of DNA damage response [37] and cellular or other viral factors in the integration process [38,39]. Cellular factors, such as Rad51, or viral factors that are involved in replication and tumorigenesis, such as UL30 (MDV polymerase), meq and vTR (viral telomerase RNA), would be exciting targets for future studies [40,41,42]. Furthermore, this system will provide insights into how the virus genome is maintained during latency, as well as subsequent reactivation. Moreover, it can be used to complement or even substitute for animal experiments. In the future, we will also use this system to investigate the integration properties of different MDV pathotypes and different MDV vaccine viruses.

## Figures and Tables

**Table 1:** Primers and probes used in this study.

Construct Name		Sequence (5′ → 3′)
eGFP in mini-F	for	GGTGACACGCGCGGCCTCGAACACAGCTG CAGGCCATGGTGAGCAAGGGCGAGG
	rev	CGTCGACCCGGGTACCTCTAGATCCGCTAGC GCTTTACTTGTACAGCTCGTCCATGCC
PCR-based probe 1	for	ATTACCTGGGGACAGCATGA
	rev	CACATCGTTTTGCCATGTTG
PCR-based probe 2	for	CCGCTTCCTATCTCAGCAGA
	rev	TCAAGCGCTTTCTCATAGGG
PCR-based probe 3	for	GAGCCAACAAATCCCCTGA
	rev	GAGGTTGGTGCTGGAATGTT
PCR-based probe 4	for	CTGTTCATGTCGGAGGTCTG
	rev	GAGGGAAGCTACGGTTCAAG
PCR-based probe 5	for	CCGACAATTATTGCCCCGTA
	rev	ATCTGGAAACATGTCCGACG
ICP4	for	CGTGTTTTCCGGCATGTG
	rev	TCCCATACCAATCCTCATCCA
	probe	FAM-CCCCCACCAGGTGCAGGCA-TAM
iNOS	for	GAGTGGTTTAAGGAGTTGGATCTGA
	rev	TTCCAGACCTCCCACCTCAA
	probe	FAM-CTCTGCCTGCTGTTGCCAACATGC-TAM
UL36	for	GACAAGCTACTACAAATTGCA
	rev	GACGTCGATTTATCTCTTAACA
	probe	FAM-AAGAACTACATCGAACGCACCCATGCTAGC-TAMRA
pp38	for	GAGCTAACCGGAGAGGGAGA
	rev	CGCATACCGACTTTCGTCAA
	probe	FAM-CTCCCACTGTGACAGCC-TAMRA
vTR	for	CCTAATCGGAGGTATTGATGGTACTG
	rev	CCCTAGCCCGCTGAAAGTC
	probe	FAM-CCCTCCGCCCGCTGTTTACTCG-TAMRA
GAPDH	for	GGTGCTAAGCGTGTTATCATCTCA
	rev	CATGGTTGACACCCATCACAA
	probe	FAM-TGTGCCAACCCCCAAT-TAMRA

for, forward primer; rev, reverse primer; FAM, 6-carboxyfluorescein; TAM, TAMRA.

## Data Availability

Not applicable.

## Supplementary Materials

The following are available online at https://www.mdpi.com/article/10.3390/microorganisms9122489/s1, Figure S1: Determination of viral gene expression levels, Figure S2: MDV integration in the MDV tumor cell line MSB-1 and in primary MDV tumor cells.
