# Supplementary material for: A Cell Culture System to Investigate Marek’s Disease Virus Integration into Host Chromosomes
Source: Microorganisms. 2021 Dec 1;9(12):2489. doi: 10.3390/microorganisms9122489 (PMC8706938; doi:10.3390/microorganisms9122489)
Supplement: Supplementary file 1 [file microorganisms-09-02489-s001.zip › microorganisms-1447096-supplementary.pdf]

Supplementary Material for:

A Cell Culture System to Investigate Marek's Disease Virus Integration into Host Chromosomes

by Y. You et al.

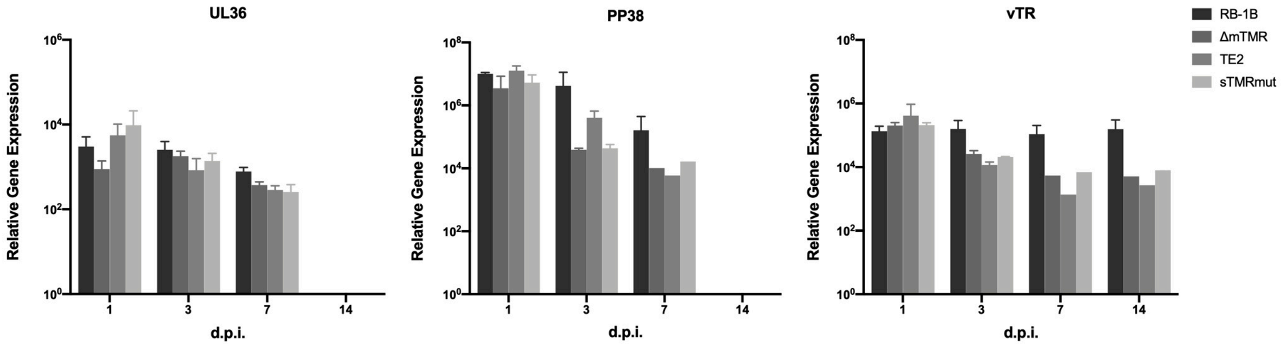

Figure S1: Determination of viral gene expression levels. Viral gene expression levels in 855-19 T cells for the early gene UL36, the late gene PP38, and the latent gene vTR were measured by RT-qPCR and normalized to the expression levels of GAPDH (n=3).

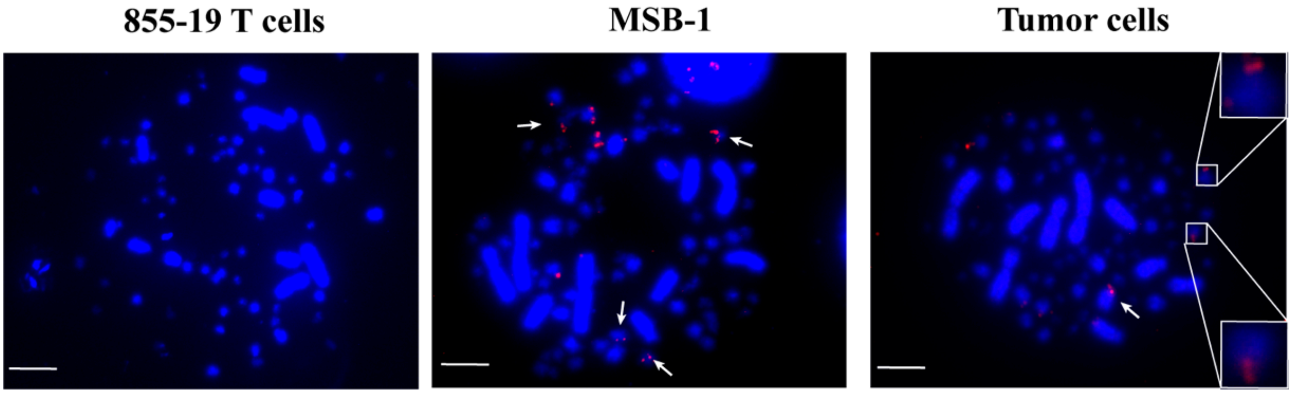

Figure S2: MDV integration in the MDV tumor cell line MSB-1 and in primary MDV tumor cells. Representative metaphase chromosomes (DAPI stain, blue) are shown along with the presence and location of integrated MDV (Cy3 streptavidin, red) in MSB-1 cell lines and MDV-induced tumor cells. Uninfected 855-19 T cells served as negative controls. Scale bars correspond to 10 μm.
